# Social Support, Coping Behaviors, and Resilience Among African Americans During COVID-19

**DOI:** 10.1093/geroni/igab046.750

**Published:** 2021-12-17

**Authors:** Sung Park

**Affiliations:** Princeton University

## Abstract

Enduring structural inequalities in the United States by race have only become more apparent during COVID-19, as African Americans experienced significant health and economic challenges that far exceeded those observed among other racial and ethnic groups. Relying on multiple nationally representative surveys, this study examines the diversity of ways in which middle-aged and older African Americans’ managed the stress and pressures associated with the pandemic. I summarize the inequities faced by African Americans before and during COVID-19, as well as trends in the utilization of social support, coping behaviors, and degree of resilience. Furthermore, this study investigates the relationship between social support and coping strategies to multiple health outcomes over time. When appropriate, comparisons to other racial and ethnic groups are made. This research underscores the importance of considering social relationships and modifiable coping behaviors when studying African American aging and well-being during times of crisis.

